# *Cryptotermes* (Isoptera, Kalotermitidae) on Espiritu Santo, Vanuatu: Redescription of *Cryptotermes albipes* (Holmgren & Holmgren) and description of *Cryptotermes penaoru* sp. n.

**DOI:** 10.3897/zookeys.148.1718

**Published:** 2011-11-21

**Authors:** Yves Roisin

**Affiliations:** 1 Evolutionary Biology & Ecology, CP 160/12, Université Libre de Bruxelles, Avenue F.D. Roosevelt 50, B-1050 Brussels, Belgium

**Keywords:** Isoptera, Cryptotermes: Vanuatu, new species

## Abstract

Complete series of two species of the phragmotic drywood termite genus, *Cryptotermes* (Isoptera: Kalotermitidae), were found on Espiritu Santo, Vanuatu. Here, I describe for the first time the soldier of *Cryptotermes albipes* (Holmgren & Holmgren), which resembles *Cryptotermes domesticus* but presents deep depressions on the head sides and vertex. The other species, here described as *Cryptotermes penaoru* **sp. n.**, comes close to *Cryptotermes tropicalis*, a species known from the tropical rainforest of northern Queensland, from which its soldier is distinguished by its more elongated head capsule.

## Introduction

The termite family Kalotermitidae, often called drywood termites, is present in all tropical, subtropical and warm temperate regions of the world (Emerson 1955; Eggleton 2000). Its abundance and diversity is however very variable. The major continental rainforests, albeit otherwise rich in termite species, are notoriously poor in Kalotermitidae. This family is better represented in drier biomes or in marginal or insular forest habitats, although its presence in rainforest canopies may have been overlooked (Roisin et al. 2006).

The family was revised at the genus level by Krishna (1961), whose classification is still largely accepted. The phylogeny of the family is poorly known, although some data are available for Australian lineages (Thompson et al. 2000). The genus *Cryptotermes* is remarkable for the strongly phragmotic head of its soldiers. It is widespread over the whole intertropical region. Several species are pests of furniture or structural wood and have been spread by man over extensive areas (Gay 1967; Evans 2011). The genus is one of the best known of the entire order, since it has been the subject of three important monographs in the past 30 years. Gay and Watson (1982) revised the Australian species, and Bacchus (1987) those from the rest of the world. More recently, Scheffrahn and Křeček (1999) published a revision of this genus in the West Indies, based on extensive recent sampling campaigns. However, the fauna of many Pacific islands remains poorly known. In Vanuatu, Gross (1975) mentions only one species, *Cryptotermes albipes*, although Gay and Watson (1982) report the presence of *Cryptotermes domesticus* as well. Collections on Espiritu Santo in 2006, under the framework of the Santo 2006 Biodiversity Survey (Bouchet et al. 2009), provided several new samples, including complete series of *Cryptotermes albipes* and an undecribed species. Here, I provide the first description of the soldier of *Cryptotermes albipes* and describe the new species as *Cryptotermes penaoru*.

## Methods

Collections took place between 9–26.xi.2006 in the Saratsi Range above Penaoru village, on the west coast of the Cumberland Peninsula, Espiritu Santo, Vanuatu, as part of a multiple-taxa survey of arthropods along an altitudinal gradient (Corbara 2011). Specimens were collected and preserved in 80% ethanol.

### Imaging

Series of optical images of specimens were taken with a Leica DFC290 digital camera mounted on a Leica Z6APO microscope, then combined by Helicon Focus software. SEM images were obtained with a Philips XL 30 ESEM.

### Measurements and their abbreviations

Measurements were taken to the nearest 0.005 mm with a Wild MMS 235 length-measuring set fitted to a Wild M6 stereomicroscope.

**Imagos:** **ED** – Eyes maximum diameter; **OD** – ocellus maximum diameter; **HLP** – Head length to postclypeus; **HWE** – Head width, maximum including eyes; **HWI** – Head width, interocular; **PW** – Pronotum width (not flattened); **T3L** – Hind tibia length; **FWL** – Forewing length (without scale).

**Soldiers**: **HLP** – Head length to postclypeus; **HLF** – Head length to frontal flange; **HLG** – Head length to genal horn; **HW** – Head maximum width; **PW** – Pronotum width; **LML** – Left mandible length (seen from below, from condyle to tip); **LW** – labrum width; **HD** – head depth, excluding postmentum; **PML** – Postmentum length; **MPW** –Maximum postmentum width; **T3L** – Hind tibia length.

### Collections and their abbreviations

ANIC Australian National Insect Collection, Canberra, ACT, Australia

MNHMMuséum National d’Histoire Naturelle, Paris, France

NHMBNaturhistorisches Museum Basel, Switzerland

RBINS Royal Belgian Institute for Natural Sciences, Brussels, Belgium

ULB Université Libre de Bruxelles, Belgium

Most of the samples presently housed in the author’s collection at the ULB will ultimately be deposited at the RBINS.

## Taxonomy

### Genus Cryptotermes

Banks, 1906

http://species-id.net/wiki/Cryptotermes

Cryptotermes – Banks, 1906: 336. Type species, by monotypy: *Cryptotermes cavifrons* Banks, 1906.
Cryptotermes Banks – Krishna 1961: 379–382, Figs 77–80 (redescription).

#### Stages.

Imagos of *Cryptotermes* can be recognized by the combination of the following criteria: left imago mandible with anterior margin of third marginal tooth clearly longer than posterior margin of first plus second marginals, and media vein bending forward to join radial sector in middle of wing or beyond.

Soldiers of *Cryptotermes* have a phragmotic head (like those of *Calcaritermes*) and all fore tibial spurs approximately equal.

### Cryptotermes albipes

(N. Holmgren & K. Holmgren, 1915)

http://species-id.net/wiki/Cryptotermes_albipes

Calotermes albipes – Holmgren N & Holmgren K, 1915: 89-90 (imago). Type locality: Maré, Loyalty Islands (New Caledonia).
Cryptotermes albipes (Holmgren, N. & K.) – Snyder 1949: 38.

#### Remarks.

The type series of this species only contains imagos. Bacchus (1987) redescribed this caste and announced the recent discovery of the soldier and its forthcoming description by J. Buckerfield of CSIRO, but this author, now deceased, shifted to another field and his contribution never appeared in print.

#### Material examined.

**Paralectotypes: NEW CALEDONIA: Loyalty Islands:** Maré Island, 17.xi.1911 (coll. Sarasin & Roux), alates only (NHMB). **Other material: VANUATU:** **Taféa:** SW Tanna Island, 28.vii.1971 (coll. K.E. Lee, det. J. Buckerfield), alates and soldiers (ANIC#15344); **Sanma:** Espiritu Santo, 28.x.1982 (coll. R.L. Paton, det. J. Buckerfield), alates and soldiers (ANIC#18883); Espiritu Santo, 10.xi.2006 (coll. det. Y. Roisin), with 1 alate, sexuals, soldiers and immatures, on forested slope above Penaoru village, alt. 300m a.s.l. (14°57.98'S, 166°38.22'E) (ULB #Santo016); *ibidem*, 12.xi.2006 (coll. det. Y. Roisin), soldiers and immatures (RBINS #15607); *ibidem*, 16.xi.2006 (coll. det. Y. Roisin), sexuals, soldiers and immatures from dead branch about 15m above ground (RBINS #15616).

#### Imago.

**Figure 1.**
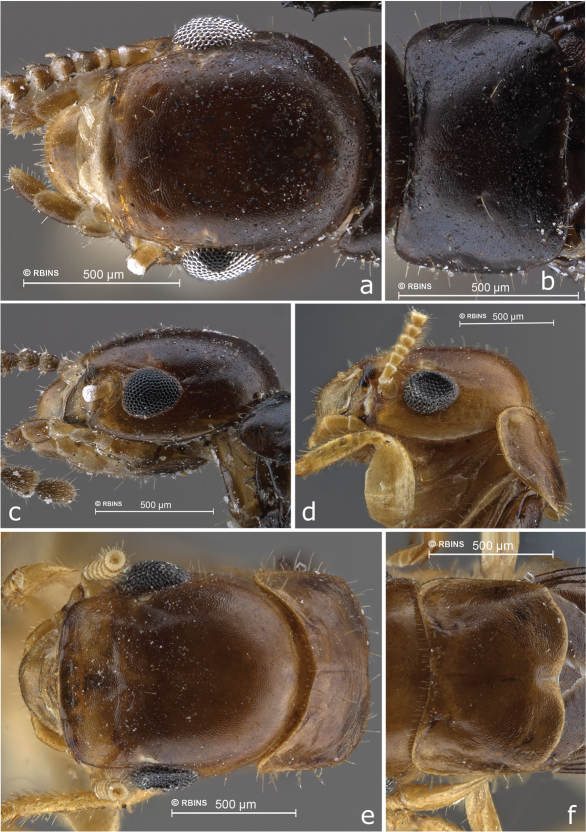
Head and pronotum of imagos **a–c** *Cryptotermes albipes* **a** head from above **b** pronotum **c** head from left side **d–f** *Cryptotermes penaoru*, paratype from colony #Santo080 **d** head from left side **e** head from above **f** pronotum.

(Figs 1a, 1c, 1e)Redescribed by Bacchus (1987: 37, figs 6–7). Pigmented parts substantially darker than described by Bacchus, as the pigmentation of Holmgren’s material faded over the years. The most remarkable feature of this species is its overall dark brown pigmentation, but with the sharp contrast between the almost white tibiae and dark brown femora.

Measurements of 6 paralectotype alates and 13 sexuals (5 alates, 8 dealates) from 4 colonies (non-type specimens between parentheses): ED: 0.270–0.310 (0.265–0.325); OD: 0.080–0.120 (0.070–0.130); HLP: 0.825–0.930 (0.835–0.885); HWE: 0.845–0.960 (0.830–0.925); HWI: 0.635–0.700 (0.620–0.665); PW: 0.755–0.885 (0.700–0.835); T3L: 0.690–0.745 (0.675–0.755); FWL: 5.06–6.11 (5.25–5.55).

Its pigmentation pattern (dark to very dark brown with pale, almost white tibiae contrasting with dark brown femora and yellow tarsi) distinguishes this species from all other *Cryptotermes* in this area.

#### Soldier.

**Figure 2.**
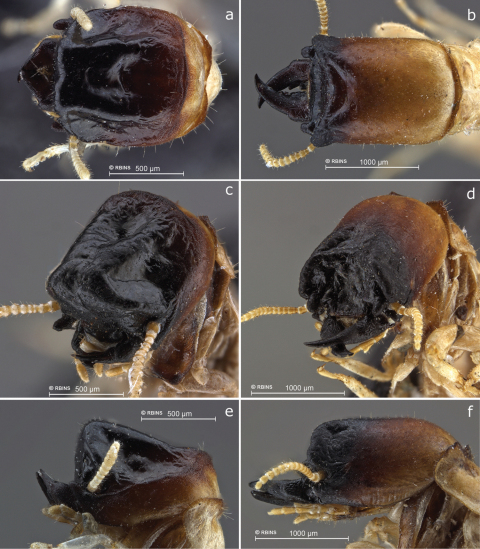
Heads of soldiers **a, c, e** *Cryptotermes albipes* **a** from above **c** oblique view from upper left front side **e** lateral view **b, d, f** *Cryptotermes penaoru*, paratype from type colony **b** from above **d** oblique view from upper left front side **f** lateral view.

**Figure 3.**
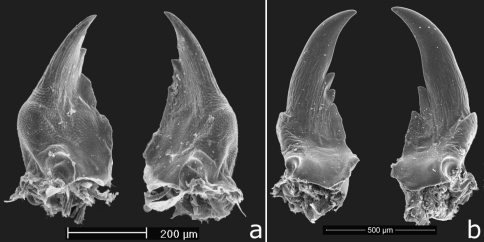
SEM pictures of soldier mandibles, from above **a** *Cryptotermes albipes* **b** *Cryptotermes penaoru* (paratype from colony #Santo080).

*(previously undescribed)* (Figs 2a, 2c, 2e, 3a)Head capsule very dark reddish brown to black. Head in dorsal view slightly convex on sides and on posterior margin; trapezoidal in profile, narrowing posteriorly. Frontal flange not raised, with medial notch. Frons flat, making an angle <90° with plane of mandibles. Genal horns well developed, pointing upwards; frontal horns reduced to low humps. Deep depression on vertex, delimited by broad, blunt crests running backwards from highest points of frontal flange. Sides of head capsule concave. Mandibles rather short and stout, with sharp cutting edge and small teeth. Antennae pale brown, of 10–12 articles. Pronotum widely and angularly notched, with thickened anterior margin.

Measurements of 6 soldiers from 5 colonies: HLP: 0.975–1.230; HLF: 0.955–1.215; HLG: 0.975–1.230; HW: 0.920–1.085; PW: 0.795–1.075; LML: 0.495–0.615; LW: 0.185–0.225; HD: 0.730–0.890; T3L: 0.525–0.625.

Vertical to overhanging frons distinguishes *Cryptotermes albipes* from all other species from the area except *Cryptotermes domesticus*. Depression in middle of vertex with conspicuous anteroposterior ridges on both sides is characteristic of *Cryptotermes albipes*.

#### Distribution and biology.

*Cryptotermes albipes* is known from the Loyalty Islands (east of New Caledonia) and Vanuatu (Espiritu Santo and Tanna). It also occurs in New Guinea (unpublished data). Its biology is poorly known. On Santo, this species was found at 300m a.s.l. in dead tree trunks on the ground, as well as in a dead branch on a living tree at a height of 15m. Colony boundaries seem rather diffuse. Several reproductive pairs can be found in the same log, and may possibly reside in the same network of interconnecting tunnels.

### Cryptotermes penaoru sp. n.

urn:lsid:zoobank.org:act:B0BB619F-6C57-4E5E-A30C-4B07B306C723

http://species-id.net/wiki/Cryptotermes_penaoru

#### Remarks.

Samples of this species were previously identified as *Cryptotermes tropicalis* Gay & Watson (Roisin et al. 2011), but further examination revealed them to belong in an undescribed species.

#### Material examined.

**Holotype, soldier: VANUATU:** **Sanma:** Espiritu Santo, 09.xi.2006 (coll. det. Y. Roisin), in standing dead wood, on forested slope above Penaoru village, alt. 100m a.s.l. (14°57.69'S, 166°37.90'E) (ULB #Santo003; RBINS #15589: type colony). **Paratypes:** alates, 1 soldier and immatures from type colony (same data as holotype); *ibidem*, 18.xi.2006 (coll. det. Y. Roisin), 1 male (dealate), 3 soldiers, immatures (ULB #Santo080).

#### Imago.

(Figs 1b, 1d, 1f) Overall colour medium brown; head, pronotum and wing scales darker; legs paler, with femora paler than tibiae; abdominal sternites palest. Wings brown, paler than tergite colour, with pimple-like nodules. Head parallel-sided, almost ciruclar behind. Eyes large; ocelli large, oval, contiguous to eyes. Antennae of 14–16 segments in alates, broken down to 7 segments in dealate of colony #Santo080. Pronotum almost as wide as head, widely concave anteriorly, with convex sides narrowing posteriorly, posterior margin biconvex. Pilosity of head and pronotum sparse and short. Wings with subcosta, radius and radial sector sclerotized, and slight sclerotization of cubital branches. Media unsclerotized, except at junction with radial sector, beyond half length of wing. Arolium present.

Measurements of paratypes: 4 alates from type colony (#Santo003) and 1 dealate from colony #Santo080 (parentheses): ED: 0.265–0.295 (0.275); OD: 0.100–0.115 (0.090); HLP: 0.950–1.000 (0.950); HWE: n.a. (0.935); HWI: 0.695–0.730 (0.705); PW: 0.810–0.910 (0.865); T3L: 0.745–0.870 (0.825); FWL: 6.82–7.15 (n.a.).

#### Soldier.

(Figs 2b, 2d, 2f, 3b)Head capsule from ferruginous posteriorly to black in frontal area. Mandibles almost black, antennae and labrum dark orange. Head quadrangular, distinctly longer than wide, with straight parallel sides and convex posterior margin. Frontal flange prominent only on sides, extending as low ridges backwards at an angle of ~45° with sagittal plane. Frons-vertex ridge concave, delimiting with posterior extensions of frontal flange a triangular depression anteriorly on vertex. Frons falling steeply on postclypeus. Frontal horns stout, prominent, blunt. Genal horns very small, blunt. Slight lateral depression and rugosity posterior to frontal flange. Eyes visible as distinct pale spots. Mandibles long, with prominent external hump at basal third. Marginal teeth small but distinct. Antennae of 10–15 articles. Pronotum widely and angularly notched, with thickened anterior margin.

Measurements of holotype, paratype from type colony [brackets] and 3 paratypes from colony #Santo080 (parentheses). HLP: 1.755 [1.675] (1.610–1.650); HLG: 1.615 [1.645] (1.560–1.580); HW: 1.250 [1.165] (1.135–1.175); PW: 1.175 [1.055] (1.035–1.095); LML: 1.025 [0.950] (0.955–0.995); LW: 0.320 [0.270] (0.260–0.325); HD: 1.025 [0.920] (0.925–0.950); T3L: 0.920 [0.875] (0.775–0.815).

This species comes clearly close to *Cryptotermes tropicalis*, from Queensland, but can be distinguished by its more elongated head.

#### Distribution, etymology and biology.

*Cryptotermes penaoru* was found in a single site in lowland forest near Penaoru village, hence its name. The type colony was collected from a small standing dead tree.

## Discussion

*Cryptotermes penaoru* clearly belongs in a group of moderately phragmotic species with low or medially indistinct frontal flange, weak to moderate lateral rugosity behind frontal flange, and relatively long mandibles with distinctive marginal teeth. This group includes the Australian species *Cryptotermes tropicalis*, *Cryptotermes primus*, *Cryptotermes austrinus*, *Cryptotermes queenslandis* and *Cryptotermes simulatus*, which form a monophyletic lineage (Thompson et al. 2000). As *Cryptotermes* species readily colonize islands and often speciate locally (Scheffrahn and Křeček 1999), the discovery of new species related to this Australian lineage in the south Pacific could be expected. The affinities of *Cryptotermes albipes* are much less obvious, as this species does not closely resemble any other one. In view of the head shape of its soldiers, *Cryptotermes domesticus* might be the best candidate to be the closest relative of *Cryptotermes albipes*. *Cryptotermes domesticus* has been widely disseminated by man, but its region of origin probably lies within southeast Asia (Evans 2011), and how far its indigenous distribution extends through Sundaland into the Papuan region and south Pacific islands is uncertain. Molecular data are badly needed to further resolve the phylogeny and phylogeography of this group.

## Supplementary Material

XML Treatment for Cryptotermes

XML Treatment for Cryptotermes albipes

XML Treatment for Cryptotermes penaoru

## References

[B1] Bacchus S (1987) A taxonomic and biometric study of the genus *Cryptotermes* (Isoptera: Kalotermitidae). Tropical Pest Bulletin No. 7. Tropical Development and Research Institute, London, U.K., 1–91.

[B2] Banks N (1906) Two new termites. Entomological News 17: 336-339.

[B3] Bouchet P Le Guyader H Pascal O (2009) The SANTO 2006 Global Biodiversity Survey: an attempt to reconcile the pace of taxonomy and conservation. Zoosystema 31: 401-406. 10.5252/z2009n3a0

[B4] Corbara B (2011) IBISCA-Santo – Biodiversity along an altitudinal gradient. In: Bouchet P Le Guyader H Pascal O (Eds). The Natural History of Santo. MNHN / IRD / PNI, Paris, France: 119-122.

[B5] Eggleton P (2000) Global patterns of termite diversity. In: Abe T Bignell DE Higashi M (Eds). Termites: Evolution, Sociality, Symbioses, Ecology. Kluwer Academic Publishers, Dordrecht, The Netherlands: 25-51.

[B6] Emerson AE (1955) Geographical origins and dispersions of termite genera. Fieldiana: Zoology 37: 465-521.

[B7] Evans TA (2011) Invasive termites. In: Bignell DE Roisin Y Lo N (Eds). Biology of Termites: A Modern Synthesis. Springer SBM, Dordrecht, The Netherlands: 519-562.

[B8] Gay FJ (1967) A World Review of Introduced Species of Termites. Bulletin No. 286. C.S.I.R.O., Melbourne, Australia, 1–88.

[B9] Gay FJ Watson JAL (1982) The genus *Cryptotermes* in Australia (Isoptera: Kalotermitidae). Australian Journal of Zoology Supplementary Series 88: 1-64. 10.1071/AJZS088

[B10] Gross GF (1975) The land invertebrates of the New Hebrides and their relationships. Philosophical Transactions of the Royal Society of London (B) 272: 391-421. 10.1098/rstb.1975.0095

[B11] Holmgren N Holmgren K (1915) Termiten aus Neu-Caledonien und den benachbarten Inselgruppen. Nova Caledonia, Zoologie 2: 85-93.

[B12] Krishna K (1961) Generic revision and phylogenetic study of the family Kalotermitidae (Isoptera). Bulletin of the American Museum of Natural History 122: 303-408.

[B13] Roisin Y Dejean A Corbara B Orivel J Samaniego M Leponce M (2006) Vertical stratification of the termite assemblage in a neotropical rainforest. Oecologia 149: 301-311. 10.1007/s00442-006-0449-5 16791633

[B14] Roisin Y Corbara B Delsinne T Orivel J Leponce M (2011) Termites in Santo: lessons from a survey in the Penaoru area. In: Bouchet P Le Guyader H Pascal O (Eds). The Natural History of Santo. MNHN / IRD / PNI, Paris, France: 128-130.

[B15] Scheffrahn RH Křeček J (1999) Termites of the genus *Cryptotermes* Banks (Isoptera: Kalotermitidae) from the West Indies. Insecta Mundi 13: 111-171.

[B16] Snyder TE (1949) Catalog of the Termites (Isoptera) of the World. Smithsonian Institution, Washington, D.C., 1–490.

[B17] Thompson GJ Miller LR Lenz M Crozier RH (2000) Phylogenetic analysis and trait evolution in Australian lineages of drywood termites (Isoptera, Kalotermitidae). Molecular Phylogenetics and Evolution 17: 419-429. 10.1006/mpev.2000.0852 11133196

